# Case Report: Chronic hepatitis E in a hematopoietic stem cell transplant recipient: The first report of hepatitis E virus genotype 4 causing chronic infection in a non-solid organ recipient

**DOI:** 10.3389/fimmu.2022.954697

**Published:** 2022-10-05

**Authors:** Zihao Chen, Junfeng Wei, Li Jiang, Dong Ying, Weikun Tian, Mengyang Zhang, Guiping Wen, Siling Wang, Chang Liu, Yingbin Wang, Ting Wu, Zimin Tang, Zizheng Zheng, Li Yan, Ningshao Xia

**Affiliations:** ^1^ State Key Laboratory of Molecular Vaccinology and Molecular Diagnostics, National Institute of Diagnostics and Vaccine Development in Infectious Diseases, School of Public Health, Xiamen University, Xiamen, China; ^2^ Department of Infectious Diseases, Henan Provincial People’s Hospital, People’s Hospital of Zhengzhou University, Zhengzhou, China; ^3^ Department of Hematology, Henan Provincial People’s Hospital, People’s Hospital of Zhengzhou University, Zhengzhou, China; ^4^ School of Life Sciences, Xiamen University, Xiamen, China; ^5^ Department of Pathology, Henan Provincial People’s Hospital, People’s Hospital of Zhengzhou University, Zhengzhou, China; ^6^ United Diagnostic and Research Center for Clinical Genetics, Women and Children’s Hospital, School of Medicine and School of Public Health, Xiamen University, Xiamen, China; ^7^ Xiang An Biomedicine Laboratory, Xiamen University, Xiamen, China; ^8^ Department of Severe Hepatology, Shanghai Public Health Clinical Centre, Fudan University, Shanghai, China; ^9^ Research Unit of Frontier Technology of Structural Vaccinology, Chinese Academy of Medical Sciences, Xiamen, China

**Keywords:** hepatitis E virus, chronic hepatitis, hematopoietic stem cells transplantation, cirrhosis, aplastic anemia

## Abstract

Hepatitis E virus (HEV) is one of the most important public health issues around the world, and chronic HEV infection has been reported in immunosuppressed individuals. This study reported a male case, with very severe aplastic anemia (AA), who developed chronic hepatitis E after hematopoietic stem cell transplantation (HSCT). Abnormal alanine aminotransferase (ALT) appeared after HSCT and persisted for twenty-nine months. The case was seropositive for anti-HEV IgG and IgM after HSCT. Twenty-two months after HSCT, HEV RNA and antigen (Ag) testing were positive and persisted for five and seven months, respectively. Positive stains of HEV Ag were present in a liver biopsy sample. HEV Ag was present in bone marrow. The individual rapidly developed liver cirrhosis and was rescued by a regimen of oral ribavirin. These factors suggested there is a risk of HEV infection in HSCT recipients.

## Introduction

Hepatitis E virus (HEV) infection usually causes acute viral hepatitis in developing and developed countries (1). In immunocompetent individuals, hepatitis E (HE) generally self-limits to acute hepatitis. However, HEV can cause ~30% mortality in pregnant women in the third trimester and also induces chronic HE infection in immunosuppressed patients (1).

Prior studies have shown that extra-hepatic manifestations, such as acute pancreatitis and musculoskeletal and hematological disorders, develop in HEV-infected individuals (2) (3). In addition, an increasing number of studies reported that individuals who received blood transfusion are at risk for HEV infection (4).

Chronic HEV infection was observed in individuals who receive immunosuppressive therapy following solid organ transplantation (SOT) or stem cell transplantation (SCT) and are HIV-positive (5). Moreover, acute and persistent HEV infection was also reported in patients with acute lymphoblastic leukemia (ALL) (6). The progressive cirrhosis caused by chronic infection has mainly been reported for genotype 3 (G3) of HEV, and occasionally for genotype 4 (G4) (5). Chronic HE (genotype unknown) in a child with ALL after SCT was reported in Canada (7). Chronic infection or fatal outcome caused by HEV G3 has been reported in some patients with hematological malignancy (8–10). Although HEV G3 has been identified as a potential burden among patients with hematological malignancy (11), including hematopoietic stem cell transplantation (HSCT) recipients, chronic infection of HEV G4 in patients undergoing HSCT has never been reported.

Here, we report a case of a 33-year-old male with very severe aplastic anemia (AA). After hematopoietic stem cell transplantation (HSCT), HEV RNA and antigen (Ag) persisted for five months and seven months, respectively. The individual rapidly developed liver cirrhosis induced by G4 (subtype 4a) chronic HE and was rescued by a regimen of ribavirin treatment.

## Case presentation

In Jan 2017, a 33-year-old male (AM-C01 AA) who reported multiple bleeding for 4 days was diagnosed with very severe AA and was treated with HSCT from HLA-matched sibling donors (MSD) until Feb 2017. A routine investigation performed prior to HSCT showed hepatitis A virus (HAV), hepatitis B virus (HBV), hepatitis C virus (HCV), HEV, cytomegalovirus (CMV), and Epstein-Barr virus (EBV) negative. For MSD, routine investigation for HBV, HCV, CMV, and EBV returned before HSCT. The conditioning regimen for HSCT consisted of cyclosporine (50 mg/kg), antithymocyte globulin (ATG, 10 mg/kg), and Mesna (30 mg/kg). Two hundred and twenty-five milliliters of peripheral hematopoietic stem cells (HSCs, 9.30×10^8^/kg mononuclear cells, and 6.23×10^6^/kg CD34^+^ cells) were given, and one hundred and fifty milliliter peripheral HSCs (6.17×10^8^/kg mononuclear cells and 3.27×10^6^/kg CD34^+^ cells) administered on the second day. Prophylaxis of graft-versus-host disease (GvHD) was comprised of ciclosporin A (CsA), methotrexate (MTX), and immunosuppressant (MMF). Acyclovir and gamma-globulin maintained anti-infection. Twenty-two days after HSCT, BM and peripheral blood analysis showed a complete hematological remission, and no GvHD was observed.

**Figure 1:**
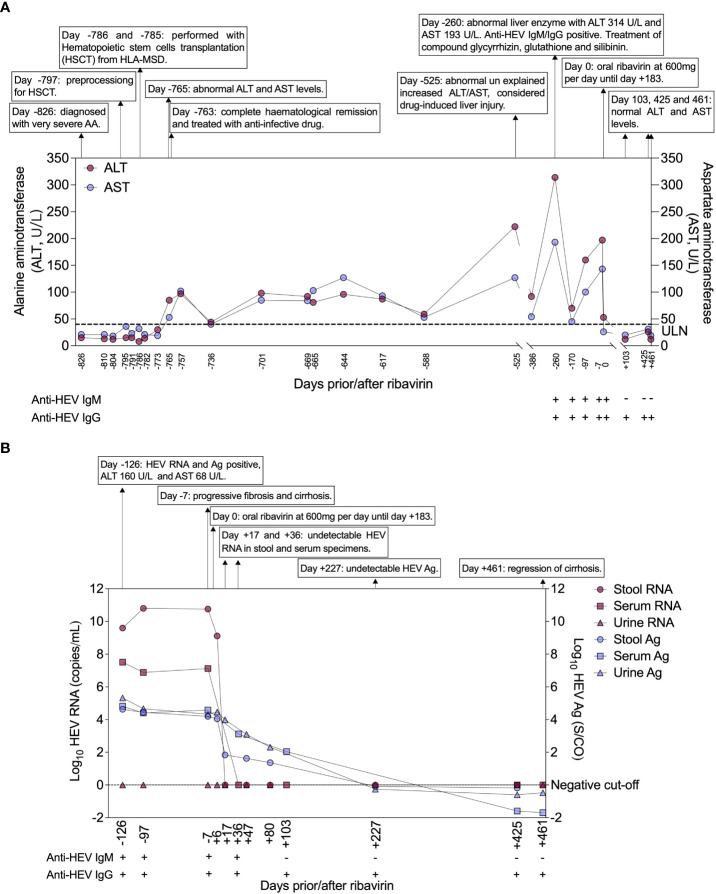
Clinical course of HEV infection prior to and after ribavirin. **(A)** Light red legend indicating ALT (circles) and light blue legend indicating AST (circles). The y-axis on the left represents levels of ALT (U/L) and the right represents levels of AST (U/L). **(B)** The light red legend indicates HEV RNA of stool (circles) and serum (squares). The light blue legend indicates HEV antigen of stool (circles), serum (squares), and urine (triangles). The y-axis on the left represents viral load of HEV (Log_10_ copies/mL) and the right represents levels of HEV Ag (Log_10_ S/CO). The x-axis of **(A)** and **(B)** are scaled to illustrate time intervals in relation to oral ribavirin. The presence and absence of anti-HEV IgM and anti-HEV IgG in serum are indicated by “+” and “–”, respectively. AA, aplastic anemia; Ag, antigen; ALT, Abnormal alanine aminotransferase; AST, aspartate aminotransferase; antigen; IgM, immunoglobulin M; IgG, immunoglobulin G.

**Figure 2:**
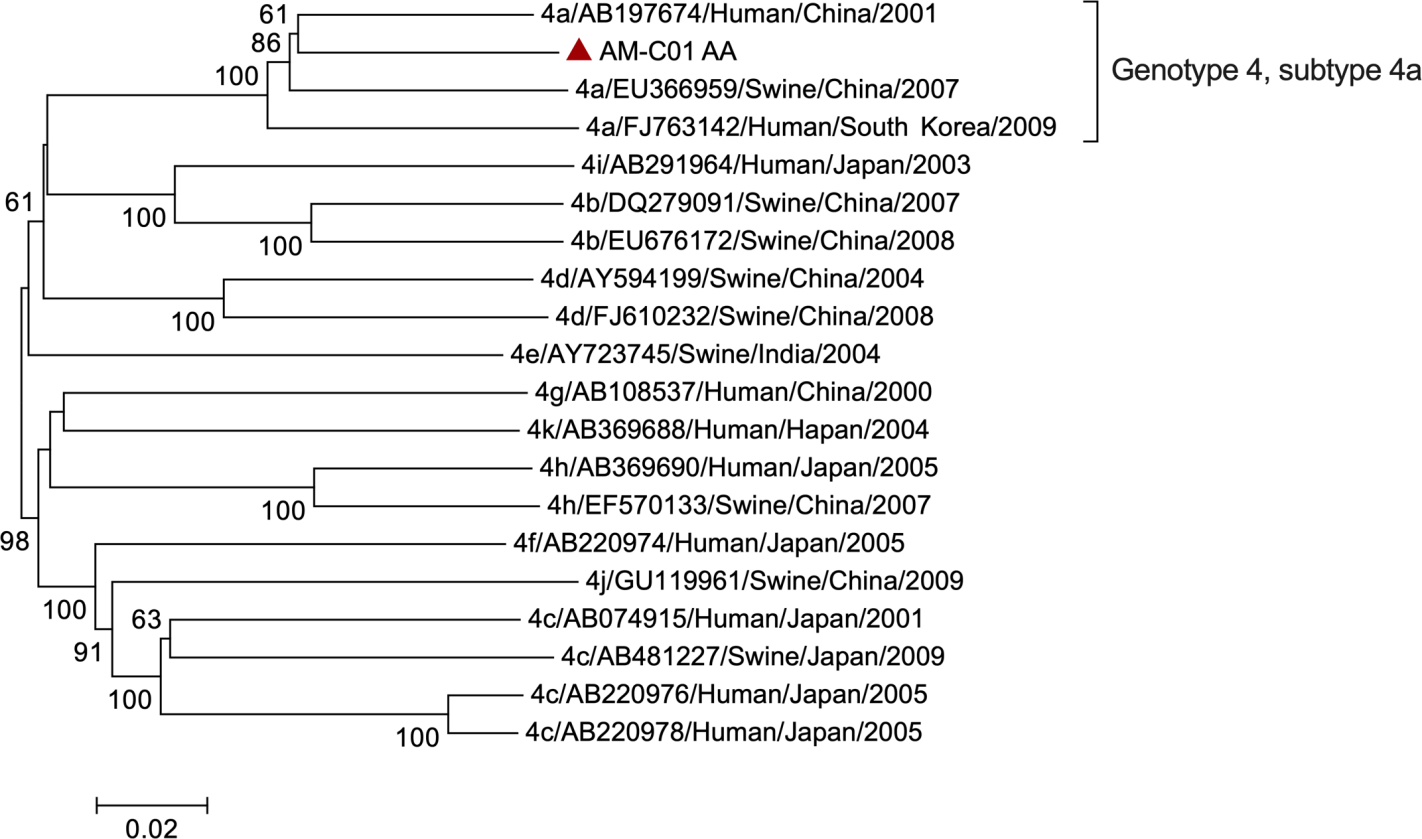
Phylogenetic tree analysis of HEV full-genome sequence in the case. The phylogenetic analysis was based on HEV full-genome. Viral genotype, GenBank accession number, virus host, country of origin, and year of detection are indicated for the reference sequences. The sequence obtained from the case is highlighted with a red triangle. The bootstrap values > 70 were shown.

Abnormal alanine aminotransferase (ALT) and aspartate aminotransferase (AST) were observed after 21 days of HSCT with 85 U/L and 53 U/L in Mar 13^rd^ 2017 (day -765), which persisted for twenty-nine months until Jul 29^th^ 2019 (day +103) (**Figure 1A**). Non-specific viral symptoms were present, such as fever. Signs of jaundice and weight loss were not observed. Though complete hematological remission was observed, the case was treated with an anti-infective drug. Upon admission to our hospital in Jul 31^st^ 2018 (day -260), abnormal liver enzymes were persistent, ALT 314 U/L, and AST 193 U/L (**Figure 1A**). Liver-directed autoantibodies were negative. Further anti-HEV IgM and IgG serological testing were performed using an enzyme-linked immunosorbent assay (ELISA) kit (Beijing Wantai, Beijing, China) according to the manufacturer’s instructions. This revealed the patient to be anti-HEV IgM and IgG positive. Treatment of hepatitis consisted of oral drugs including compound glycyrrhizin, glutathione, and silibinin. HEV RNA detection was performed using real-time RT-PCR using QIAGEN OneStep RT-PCR Kit (Qiagen, Hilden, Germany) as previously reported (12), and HEV Ag-ELISA assay (Beijing Wantai, Beijing, China) was performed on serum and stool samples. Follow-up samples in serum and stool revealed HEV RNA and Ag positive in Dec 12^nd^ 2018 (day -126), ALT 160 U/L, and AST 68 U/L (**Figures 1A, B**). Serological testing revealed anti-HEV IgM and IgG to be persistent (**Figure 1B**). Viral RNA was extracted from the patient’s stool supernatant using QIAamp Viral RNA Mini Kit (Qiagen, Hilden, Germany), and viral genome library was built for the sample based on next-generation sequencing (NGS) results (GenBank no. OP185389). The sequence analysis based on a full-length genome revealed that the patient was infected with HEV G4, subtype 4a (**Figure 2**).

**Figure 3:**
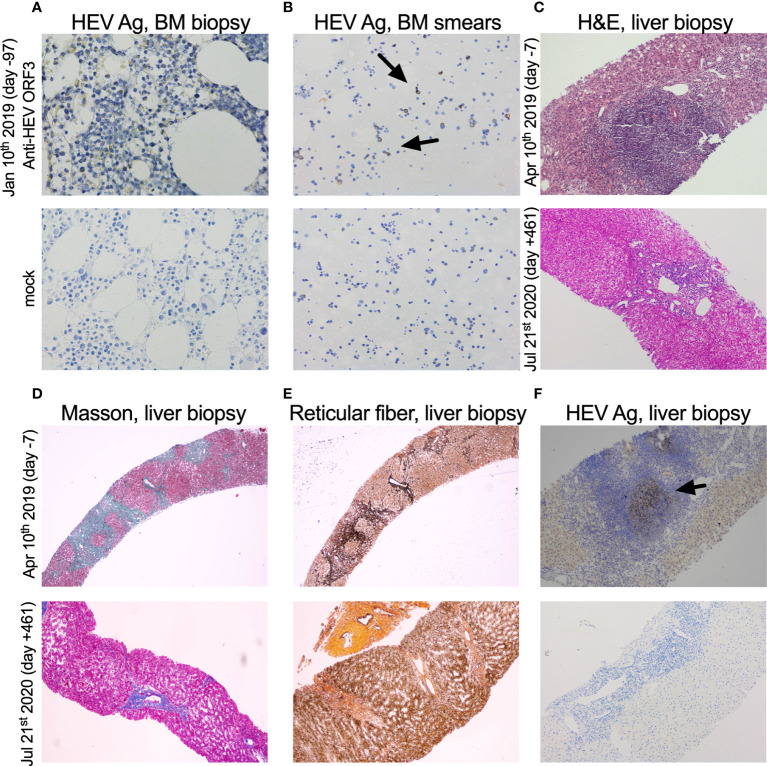
Immunohistochemical detection of HEV antigen and pathological analysis of bone marrow and liver biopsy samples. **(A, B)** HEV antigen (HEV Ag) detection of bone marrow (BM) biopsy (×400) and smears (×200). BM biopsy and smears (Jan 10^th^ 2019, day -97) collected from the case were shown. **(C)** H&E of the liver biopsy (×100). **(D, E)** Masson trichrome and reticular fiber staining of liver biopsy (×40). **(F)** HEV Ag detection of liver biopsy (×100). Liver biopsy samples prior to and after oral ribavirin (Apr 17^th^ 2019, day 0) were shown above (Apr 10^th^ 2019, day -7) and below (Jul 21^st^ 2020, day +461), respectively. The time of sample was designated on the left side of each row of pictures. HEV-specific anti-ORF3 1E6 antibodies (Millipore, Temecula, CA) were used in IHC of HEV Ag.

Bone marrow (BM) biopsy was performed for the case in Jan 10^th^ 2019 (day -97) and moderate positive stains of HEV Ag were found in BM cells detected by immunohistochemistry (IHC) (**Figure 3A**). To further investigate the presence of HEV in BM, IHC of HEV Ag was performed in BM smears, and positive stains were detected (**Figure 3B**). HEV RNA (6.72×10^2^ copies/mL) and HEV Ag (S/CO 11919.4) was detected in BM fluid. These results corresponded to a recent study reported by Wang et al. (13). They demonstrated the presence and persistence of HEV RNA and Ag in human BM and BM tropism of HEV both G3 and G4.

**Figure 4:**
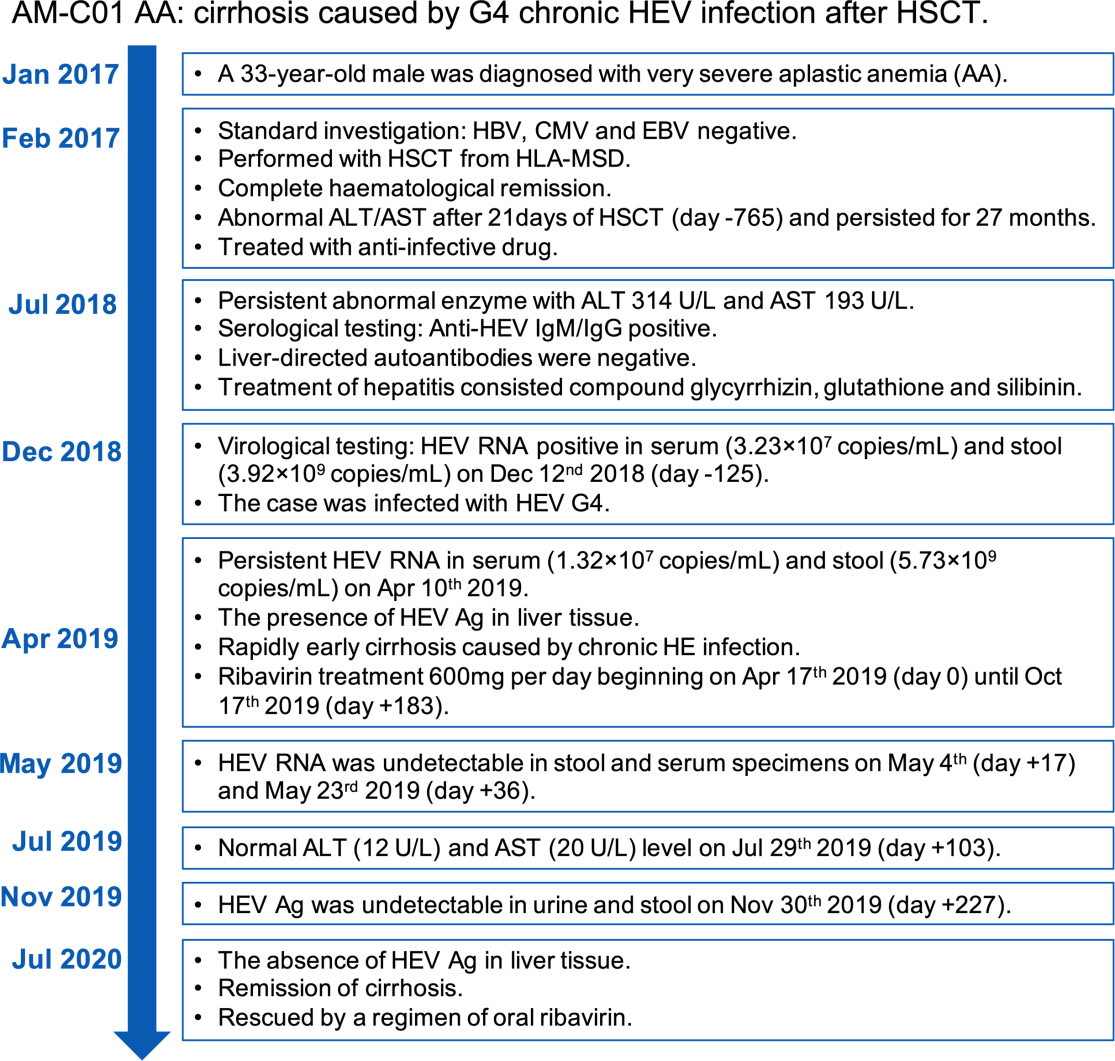
Overview of the case’s diagnosis, management, and follow-up process and timelines of major clinical events.

Hematoxylin and eosin staining (H&E) of a liver biopsy sample collected on Apr 10^th^ 2019 (day -7) showed a large number of focal lymphocytes aggregates, severe interface hepatitis, and lobular architectural disruption (**Figure 3C**). A biliary pattern was shown in the liver biopsy, and increased fibrosis was present in the portal area, with a medium amount of lymphocytes infiltrate (**Figure 3D**). Collapsed reticular fibers scaffold was shown in hepatic lobule (**Figure 3E**). The histopathological findings and the presence of HEV Ag in liver tissue were compatible with chronic HE (**Figure 3F**). Beginning on Apr 17^th^ 2019 (day 0), treatment of chronic HE consisted of oral ribavirin at a dose of 600 mg per day until Oct 17^th^ 2019 (day +183). No main adverse event (AA) was observed during the 6 months of antiviral therapy. Samples did not detect HEV RNA in stool and serum specimens at day +17 and +36 and HEV Ag was undetectable in urine and stool specimens on Nov 30^th^ 2019 (day +227) (**Figure 1B**). The case was found with a normal ALT and AST at 12 U/L and 20 U/L on Jul 29^th^ 2020 (day +103) (**Figure 1A**). On Jul 21^st^ 2020 (day +461), a liver biopsy showed mild interface hepatitis, reduced portal and lobular inflammation, with few lymphocytes infiltrate and regression of cirrhosis and architectural disruption (**Figures 3C–E**). The absence of HEV Ag in liver tissue was detected by IHC (**Figure 3F**). The overview of the case’s diagnosis, treatment, and follow-up process and timelines of major clinical events are summarized in **Figure 4**.

## Discussion

Chronic HEV infection is most often observed in immunosuppressive individuals receiving SOT, with 57.1% to 65.9% of HEV-infected individuals developing chronic hepatitis in previous studies (14, 15). HEV infection appears to be a rather rare risk in HSCT recipients (non-SOT) (16, 17). However, as evidenced by a retrospective cohort of 328 patients with 2.4% (8/328) HEV incidence rate, chronic HEV infection was also observed in HSCT recipients, with 62.5% (5/8) patients of HEV G3 infection developing chronic hepatitis (18). Carré et al. reported the first case of Ph ALL with a HEV-induced fatal event with G3 after HSCT (19).

The European Center for Disease Prevention and Control (ECDC) expert panel weighed that the minimum criterion for HEV diagnosis is anti-HEV IgM/IgG positive. Currently, the most widely used test for diagnosing HEV infection is a serological assay that detects anti-HEV IgM, which is a rapid, cost-effective, and clinically easy-to-use method. Recently, a study reported by Riveiro-Barciela et al. revealed unexpected long-lasting anti-HEV IgM positivity: anti-HEV IgM was detected positively in 56% (Mikrogen) and 24% (Wantai) of patients after a median 34 month follow-up (20). In addition, Abravanel et al. revealed low sensitivity for performance of anti-HEV IgM assay in patients receiving immunosuppressive therapy (21), similar to previous studies (15, 22). In our study, humoral response against HEV was observed since Dec 12^nd^ 2018 (day -126), and anti-HEV IgG titer had a maximum of 2.9 WHO unit per milliliter (Wu/mL). Though our case showed a serological reaction, it suggested that anti-HEV IgM testing has some limitations in diagnosis of HEV infection.

Prevention, management, and treatment of HEV infection in HSCT recipients should be further studied. In Sweden and Europe, 25% to 47.6% of HSCT recipients gradually developed chronic HEV infection and death with ongoing HE occurred in 10% of chronic HEV-infected patients (11, 23). Management of reduced immunosuppressive therapy in HSCT recipients with HEV infection was preferably recommended by some reports (24, 25). Some studies revealed GvHD and death associated with immunosuppressive alleviation (11, 26). Consequently, ribavirin should be considered firstly and early in HSCT recipients with HEV infection instead of immunosuppressive alleviation. Red cell distribution width (RDW), neutrophil to lymphocyte ratio (NLR), and RDW to lymphocyte ratio (RLR) are capable of predicting the development of liver failure during treatment (27).

The first case of chronic HEV infection in patients with hematological diseases was reported by 7 in Canada, in which chronic HEV G3 (subtype 3a) infection was observed in a child with acute lymphoblastic leukemia after HSCT (7). In 2014, Geng et al. reported the first chronic HE case with lymphoblastic leukemia caused by HEV G4 (6). In another two studies, four cases of chronic HE caused by G4 in renal transplant recipients were reported by Sridhar et al. and Wang et al. (28, 29). Future research into G4 chronic HEV in SOT or non-SOT recipients should be considered.

In this study, HEV-G4-induced chronic HE was observed in a male patient with severe AA. Although Mallet et al. reported that patients who were treated with ribavirin for three months cleared HEV and achieved sustained virological response (SVR) rates of ~80% (30), HEV Ag remained positive in specimens at 103 days after treatment in our case. The extended duration (six months) for antiviral therapy was considered. One study emphasized that, although serum and feces may be negative for viral RNA due to inhibition of HEV replication by drug (ribavirin and others), viral Ag can still be detected, suggesting that the virus may enter liver cells under a low replication status (31). The liver biopsy indicated fibrosis and cirrhosis in the patient before antiviral treatment. In the early stage of liver cirrhosis, most liver cells and tissue maintain a normal physiological status, and timely treatment may effectively reduce liver inflammation. Indeed, liver function could be partially restored after the removal of pathogenic factors in the patient.

In summary, we report the first case, as far as we know, of G4 HEV-induced chronic infection in a non-SOT recipient. It is also the sixth reported case of chronic HE caused by G4. The study suggests that HEV G4-infected patients may be at risk of developing chronic hepatitis if they are also HSCT recipients.

## Data availability statement

The datasets presented in this study can be found in online repositories. The names of the repository/repositories and accession number(s) can be found in the article/supplementary material.

## Ethics statement

The studies involving human participants were reviewed and approved by School of Public Health, Xiamen University. The patients/participants provided their written informed consent to participate in this study.

## Author contributions

Conceptualization, ZT, ZZ, LY, and NX. Methodology, ZC, JW, LJ, DY, WT, MZ, SW, CL, YW, and TW. Resources, JW, LJ, MZ, YW, TW, ZT, ZZ, LY, and NX. Writing—original draft preparation, ZC and ZT. Writing—review and editing, ZC, GW, CL, and ZT. Funding acquisition, GW, ZT, ZZ, and NX. All authors contributed to the article and approved the submitted version.

## Funding

This research was funded by the National Natural Science Foundation of China, grant numbers 82071783, 82171746 and 82001757, the Major Science and Technology Project for Significant New Drugs Creation, grant number 2018ZX09303005-002, and the CAMS Innovation Fund for Medical Sciences, grant number 2019RU022.

## Conflict of interest

The authors declare that the research was conducted in the absence of any commercial or financial relationships that could be construed as a potential conflict of interest.

## Publisher’s note

All claims expressed in this article are solely those of the authors and do not necessarily represent those of their affiliated organizations, or those of the publisher, the editors and the reviewers. Any product that may be evaluated in this article, or claim that may be made by its manufacturer, is not guaranteed or endorsed by the publisher.
